# Critical pore size for micropore filling in coal samples with different rank coals

**DOI:** 10.1371/journal.pone.0264225

**Published:** 2022-03-11

**Authors:** Lin Hong, Wenjing Wang, Dameng Gao, Wentong Liu

**Affiliations:** 1 College of Safety Science & Engineering, Liaoning Technical University, Huludao, Liaoning, China; 2 Key Laboratory of Mine Thermodynamic Disaster & Control of Ministry of Education, Huludao, Liaoning, China; Universiti Malaysia Pahang, VIET NAM

## Abstract

The objectives of this study were to explore the occurrence and migration of coalbed methane in coals of different ranks and reveal the microscopic reservoir space and the mechanism of coalbed methane. To meet these objectives, this study selected six coal samples of different coal ranks for low-pressure N_2_ adsorption experiments, explored the critical pore filling characteristics of packed N_2_ molecules in the coals, and analyzed the low-pressure N_2_ adsorption/desorption experimental isotherms using the DFT method and DA equation based on the micropore filling theory. Finally, the critical filling pressure and pore size range for micropore filling were determined, and the analysis results were verified by combining the Langmuir, DA, and BET equations. The results showed that, from low to high coal rank, the N_2_ adsorption/desorption isotherms of the coal samples transition from type Ⅱ to type Ⅰ. The proportion of N_2_ molecules in low-rank coals in the form of micropore filling and monolayer adsorption was higher than that in high-rank coals. The critical pressure and critical pore size for micropore filling exhibited U-shaped correlations with the coal rank. Low-rank coals (lignite and long flame coal) were gradually filled in the relative pressure range *P/P*_*0*_ ≈ 1E-4–0.03, and medium- and high-rank coals (gas coal, 1/3 coking coal, lean coal, and anthracite) were filled in the relative pressure range *P/P*_*0*_ ≈ 1E-4–0.01; the corresponding critical pore size ranges were 1.7–2.19 and 1.61–2.00 nm, respectively.

## Introduction

The nanopore characteristics of coal seams are key factors determining their gas-bearing properties. However, coal has a low diffusion capacity [1], because of which the occurrence of coalbed methane (CBM) is mainly in the pores of coal in the packed, adsorbed, and free states. Although the number of micropores in coal is high [2], given the limited adsorption space, the adsorption in the micropores is via the filling of the micropore volume rather than surface covering. Studying the critical filling state of the filling gas in coals has important theoretical significance for distinguishing the different adsorption forms of CBM.

Currently, the quantitative analysis of the pore structure characteristics (pore size, distribution, and connectivity) mainly relies on high-pressure mercury injection, gas adsorption (N_2_, CO_2_, Ar, and CH_4_), low-field nuclear magnetic resonance (NMR), small-angle scattering (SAXS, SANS), and other experimental techniques [3]. The physical adsorption method is widely used owing to its economy, high efficiency, and ease of operation. However, the size of the pores through which various gas molecules enter is different. Scholars have conducted several studies and have largely concluded that the mercury intrusion method is mainly suitable for studying the pore structure characteristics of macropores (>50 nm) in porous media, the low-pressure N_2_ adsorption experiment is suitable for mesopores (2–50 nm), and the low-pressure CO_2_ adsorption experiment is suitable for micropores (<2 nm) [4–13]. This is determined by multiple factors such as the molecular activity, molecular structure, molecular diameter, saturated vapor pressure, and experimental temperature.

Scholars at home and abroad have conducted research on the use of the physical adsorption method in exploring the influence of the pore structure characteristics on the adsorption performance. Thommes et al. [4] evaluated the surface chemistry and pore structure of porous materials using physical adsorption (H_2_O, Ar, N_2_, and CO_2_), XPS, and TPD-MS methods. Wang et al. [14] established an analytical method to more accurately determine the coal surface area and pore size distribution through experiments. Sing [15] used nitrogen adsorption to characterize porous materials, explaining the possible problems encountered in physical adsorption experiments. Zhang et al. [16] further discussed the measurement accuracy of physical adsorption in microporous and mesoporous materials and the characteristics of nanoscale pores and macromolecular structures of coal samples at different temperatures and pressures [17]. They used the low-pressure N_2_, CO_2_ adsorption, and infrared spectroscopy to identify the pore and macromolecular structure of the coal samples [18, 19]. To explore how the coal pore structure affects the occurrence of CBM, Wang et al. [20] studied the influence of coal pore structure changes on the gas adsorption characteristics under high temperature and high pressure. Hong et al. [21] studied the relationship between coal pore adsorption capacity and gas outburst, and proposed a new method to distinguish coal and gas outbursts. Several studies have shown that an in-depth quantitative characterization of the nanopores in coal is conducive for exploring the occurrence and enrichment mechanism of CBM.

Coal is a complex porous media material with a relatively developed microporous structure, which is closely related to factors such as the degree of deterioration, moisture, temperature, confining pressure, mineral composition, and micro-organisms. Absorbed and packed methane accounts for 80%–90% of the methane in coal reservoirs [22, 23]. Yakovlev et al. [24] selected microporous adsorbents for CO_2_ adsorption experiments and found that the adsorption heat is closely related to the temperature in the high microporous filling area or at high temperatures, the microporous adsorbents expand, and the adsorption heat in the low filling area gradually stabilizes. The study found that macropores mainly contribute to the total pore volume of coal samples, while the proportion of micropores in the total specific surface area is the highest [25–27].

To describe the micropore adsorption performance of porous materials, the theory of filling micropores (TVFM) proposed based on the Polanyi adsorption potential theory is mainly used. The smaller the pores, the stronger the interaction between the micropores and the adsorbate in the solid, and the easier the filling process in the microporous area [28]. The DFT method based on the density functional theory is different from the conventional adsorption theory (the scope of application is limited, and the experimental isotherm is described by an isotherm containing a few parameters). This method can correlate the adsorption isotherm with the microscopic characteristics of the system and is suitable for various types of micropores and mesopores with unimodal and multimodal pore size distributions. Therefore, this theory is widely used in studying microporous structure characteristics. To explore the specific behavior of the molecules in micropores, the adsorption characteristics of N_2_, Ar, and CO_2_ in different microporous materials were analyzed, and the DA equation was used to characterize and calculate the adsorption potential distribution, pore size distribution, and micropore filling rate of the microporous materials [29–31]. The TVFM theory was applied to modify the description of adsorption thermodynamics [32], the main occurrence of methane in coals was explored [33, 34], and the TVFM theory was extended to the study of ultra-microporous adsorption. The characteristic capacity of ultra-microporous adsorption could be enhanced, indicating that this effect is due to the large potential field overlap relative to the pore wall and the reduction in the surface adsorption film in terms of the volume of the filled micropores [35]. The above research proves the wide applicability and accuracy of the TVFM theory and DFT method in micropore research.

In summary, most existing studies analyzed the characteristics of the microporous structure of coal and explored the adsorption characteristics of adsorbed and packed gases. There are relatively few studies on the transition stage from the packed state to the adsorbed state for the pores in coals. The use of the micropore filling theory to study the adsorption performance in the micropore filling stage is mostly a qualitative approach, and the boundary of the different occurrence states of the adsorbate gas in coal is not clearly defined.

To this fill research gap, this study applied the Dubinin–Astakhov (DA) method of the micropore filling theory and the DFT method to quantitatively analyze and verify the critical pore filling pressure and critical pore size range for micropore filling based on low-pressure nitrogen adsorption experiments. To thoroughly explore the occurrence state of CBM in coal samples with different rank coals, correct the errors in the previous analysis of the pore structure by monolayer adsorption and multilayer adsorption, explore the transition stage of the CBM filling state and adsorption state, the pore structure and adsorption characteristics in the coals were accurately analyzed. The results provide important theoretical guidance for studying the occurrence and migration of CBM, its adsorption mechanism, and the prevention and control of coal and gas outbursts.

## Experimental samples and methods

### Sample overview

In the experiment, coal samples were selected for the analysis, including lignite, long flame coal, gas coal, 1/3 coking coal, lean coal, and anthracite. In accordance with ISO 18283: 2006, we prepared coal samples with a particle size range of 0.18–0.25 mm (60–80 mesh) and samples with a particle size <0.18 mm. For the latter group, the test-air-dry-based moisture (*M*_*ad*_), dry base ash (*A*_*ad*_), dry ash-free base volatile matter (*V*_*daf*_), and air-dry-based fixed carbon (*FC*_*ad*_) were obtained in accordance with the ISO 11722: 1999, ISO 1171: 1997, and ISO 562: 1998 industrial analysis method. Table 1 lists the test results.

**Table 1:** Basic parameters of experimental coal samples.

Sample	Coal rank	*M*_*ad*_/%	*A*_*ad*_/%	*V*_*ad*_/%	*FC*_*ad*_/%
**PH**	Lignite	16.84	11.59	34.72	36.86
**JB**	Long flame coal	5.21	14.20	24.93	55.67
**LH**	Gas coal	2.14	26.06	29.63	42.17
**HG**	1/3 Coking coal	1.62	6.78	33.08	58.52
**CV**	Lean coal	1.25	9.17	13.24	76.34
**YW**	Anthracite	1.46	9.78	10.55	78.21

### N_2_ adsorption/desorption experiment at 77 K (LPGA-N_2_)

**Fig 1:**
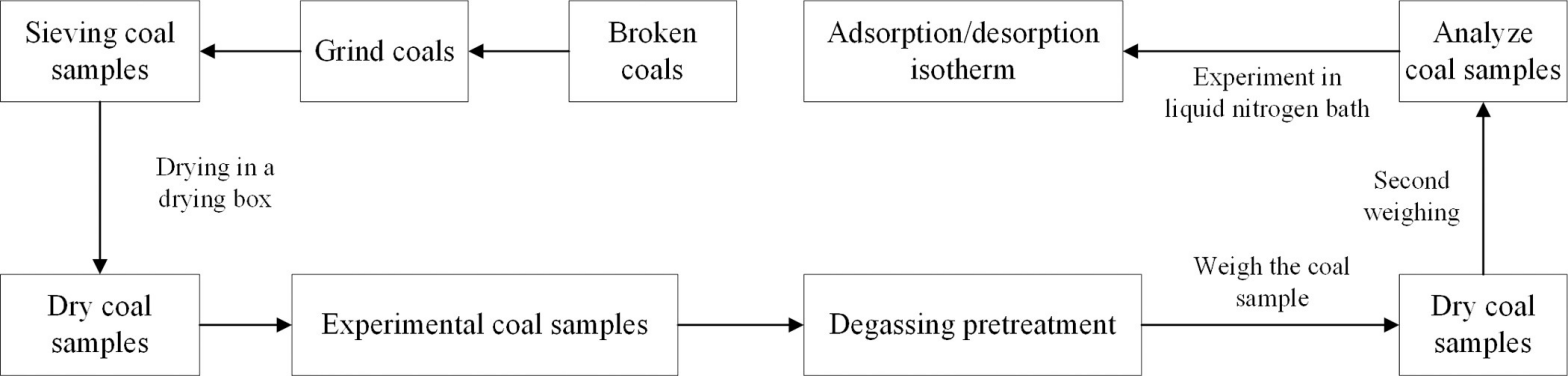
Experimental flowchart.

In each group of experiments, 60–80 mesh coal samples were selected to avoid analyzing the microscopic pore characteristics with different sample sizes. Moreover, to prevent the samples from being sucked into the internal pipes of the instrument, it is not appropriate to use fine powder samples with too small particle sizes. High-purity (99.999%) nitrogen was selected as the adsorbent gas. Before starting the experiment, the coal samples were placed in a drying box for 8 h at 373 K to prevent excessive moisture and impurities in the samples from damaging the turbo molecular pump. Subsequently, 2–3 g of the coal sample was weighed and placed in the sample tube and installed on the degassing station of the automatic physical/chemical adsorption analyzer (ASAP2020, Micromeritics, American). Heating and degassing pretreatment was then performed at 393 K for 12 h to remove the adsorbed moisture, impurities, and other volatile gases in the samples. After degassing, when the temperature dropped to room temperature, the sample tube was removed from the degassing station and quickly weighed. The sample tube was then installed in the analysis station for the low-pressure nitrogen adsorption experiment (LPGA-N_2_) at the liquid nitrogen temperature (77 K) and relative pressure *P/P*_*0*_ = 1E-6–0.995 to determine the adsorption/desorption isotherm of the coal samples. In the low-relative-pressure region (*P/P*_*0*_ = 1E-6–0.01), the low-pressure gas injection mode was applied, and the single gas injection volume was set according to the actual situation. After the adsorption equilibrium was reached, the pressure value was automatically recorded, and the above process was repeated until *P/P*_*0*_ reached 0.01. When *P/P*_*0*_ reaches 0.01, the instrument entered the constant-pressure adsorption mode. The adsorption equilibrium pressure point was set, N_2_ was continuously injected into the sample tube, and whether the pressure *P* in the tube had reached the adsorption equilibrium was monitored in real time within the equilibration interval. The equilibration interval was set to 30 s (the pressure was repeatedly monitored at an interval of 30 s). In addition, the independent *P*_*0*_ (saturated vapor pressure) sensor can quickly analyze and provide the *P*_*0*_ value under the experimental conditions for continuous *P*_*0*_ monitoring. The isothermal jacket provided accurate and stable low-pressure control for the cooling area during the experiment, ensuring that the temperatures of the *P*_*0*_ tube and sample tube were the same. Fig 1 shows the experimental flowchart.

## Experimental results and analysis

### N_2_ adsorption/desorption isotherms

**Fig 2:**
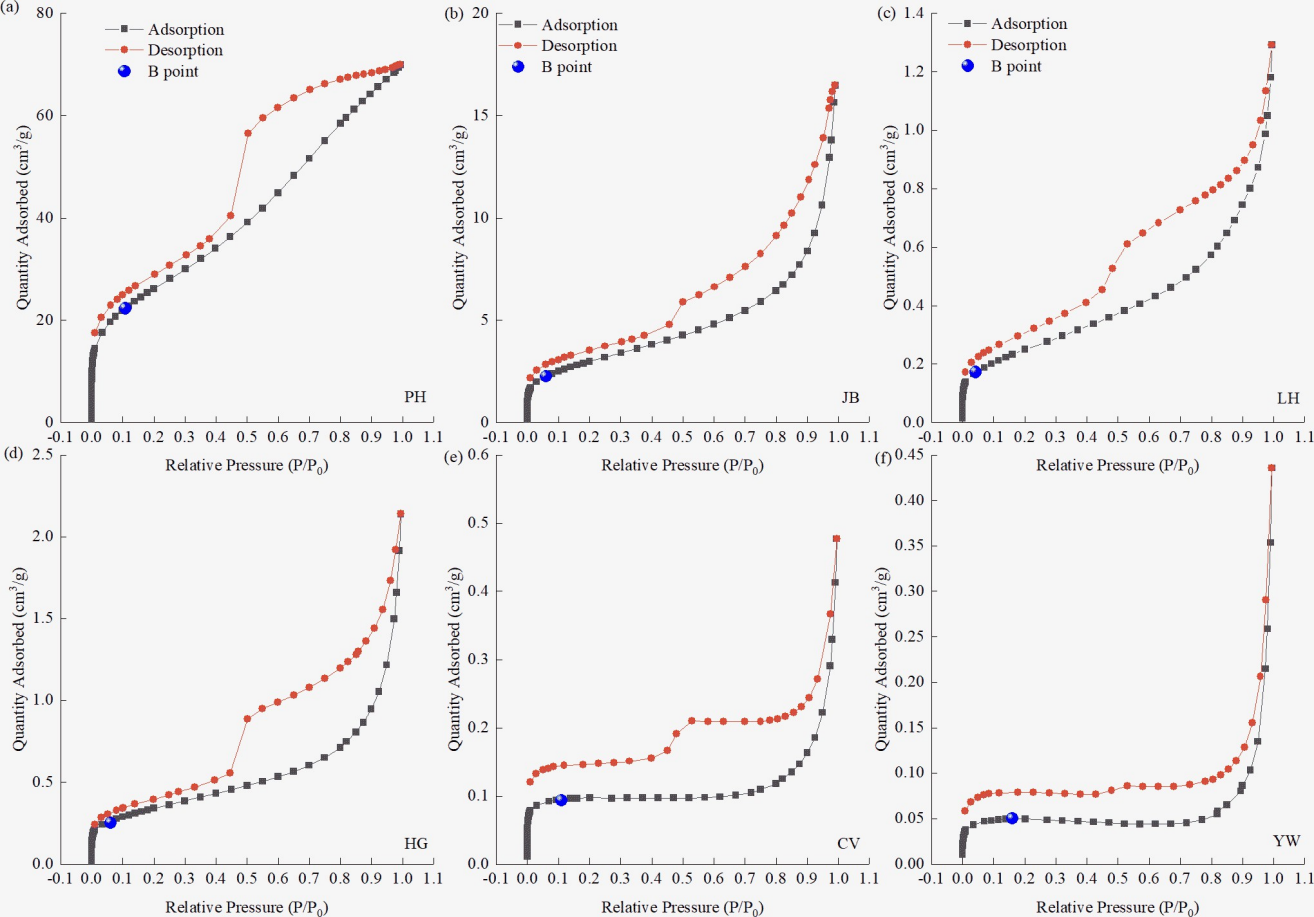
Adsorption/desorption isotherms of coal samples of different coal ranks. (a) PH Sample, (b) JB Sample, (c) LH Sample, (d) HG Sample, (e) CV Sample, (f) YW Sample.By analyzing the types of hysteresis loops of the six coal samples, the N_2_ adsorption/desorption isotherm of the PH coal sample can be approximated to H2-type hysteresis loops, indicating that the coal samples may contain some ink bottle holes. The JB, LH, and HG coal samples belong to H3-type hysteresis loops, indicating that the coal samples are mainly composed of non-uniform slit holes formed by the accumulation of flake particles. The CV and YW coal samples belong to H4-type hysteresis loops, similar in shape to H3-type. However, samples showing H4-type typically contain uniform and narrow slit pores, indicating the presence of non-rigid aggregates of micropores and flaky particles in the coal sample. Except for the PH coal sample, the desorption branch of the other five coal samples suddenly dropped when *P/P*_*0*_ = 0.42–0.5, approaching the adsorption branch; however, there was no closure point in the adsorption/desorption isotherm hysteresis loop. This phenomenon is typically due to solid swelling (intercalation phenomenon) [28] or due to the nature of the sample itself. The desorption branch deviations of the CV and YW coal samples were more evident than those of the other coal samples. This is attributed to the expansion of the interlayer distance due to adsorption. The interlayer distance is several times the molecular diameter, which is close to the micropore size, and the interlayer is then entered. The gas molecules are difficult to desorb; therefore, the isotherm does not close even under a very low relative pressure.

The coal samples selected for the study were typical porous solid materials. As per the pore classification standards recommended by IUPAC, pores can be divided into three types: micropores (<2 nm), mesopores (2–50 nm), and macropores (>50 nm). Fig 2 shows the N_2_ adsorption/desorption isotherms of six coal samples with different coal ranks. According to the latest nine classification standards of the adsorption isotherms given by IUPAC, the PH, JB, LH, and HG coal samples have a type Ⅱ(b) isotherm. This shows that the coal samples contain different numbers of micropores, mesopores, and macropores. The CV and YW coal samples have a type Ⅰ(b) isotherm. Their isotherms rise steeply and reach the plateau at low *P/P*_*0*_, indicating that the pore size changes little, and the micropore filling occurs mainly at this time. Therefore, from low-rank to high-rank coals, the adsorption isotherm of the coal samples transitions from type Ⅱ isotherm to type Ⅰ isotherm. The number of micropores in the coals increases, and the micropore filling process is relatively prolonged. The most evident difference lies in the isotherm shapes between the PH and YW coal samples, and the difference in the pore structure of coals is the largest.

**Fig 3:**
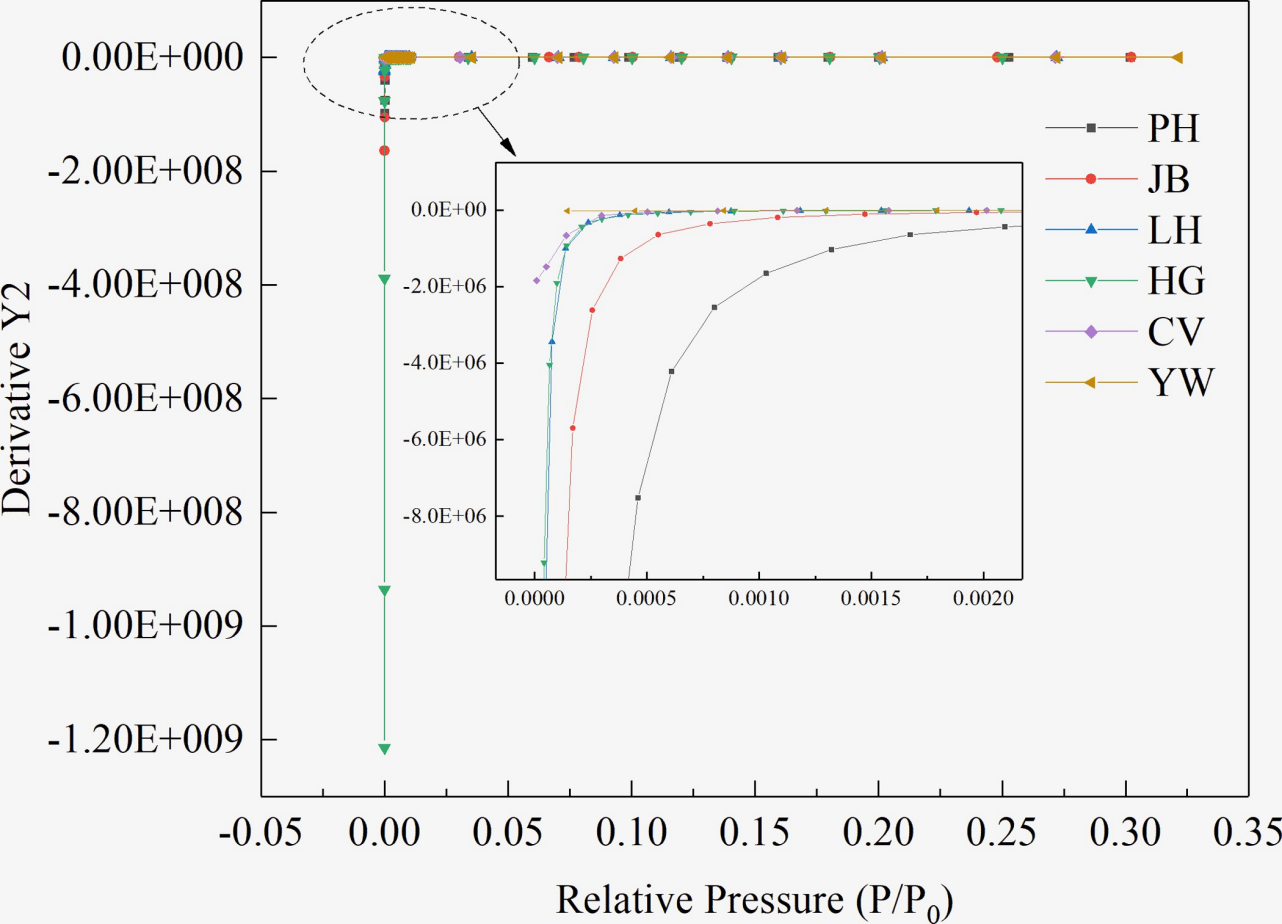
Curvature curves of the adsorption isotherms of coal samples of different coal ranks.

Due to the occurrence of micropore filling in the extremely-low-relative-pressure (*P/P*_*0*_ = 1E-6–0.01) region, the adsorption capacity increased sharply, indicating the presence of molecular-sized micropores in the six coal samples. However, there are differences in the pressure range of the filling and the growth rate of the adsorption capacity, indicating that the number and size of the micropores in the six coal samples are different. After the micropores are filled, the molecular-sized pores (regions with higher potential energy) are filled, and a single-layer covering is carried out on the solid surface (regions with high potential energy), that is, the gas adsorbed on the solid surface is only one molecule thick. When the solid surface is covered with a layer of adsorbed molecules, the atomic force field on the surface of the adsorbent is saturated, making this process a monolayer adsorption. Emmet and Brunauer [36] referred to the starting point of the second straight part of the isotherm as the inflection point B, and the ordinate *Q*_*B*_ of point B as the saturated adsorption capacity of the monolayer *Q*_*m*_. Through studies and comparisons, it was found that *Q*_*B*_ and *Q*_*m*_ of most type II isotherms are similar; the error is within 10% after replacement. As shown in Fig 2, the point B of the six coal samples is in the *P/P*_*0*_ range of 0.04–0.16. From low-rank coal to high-rank coal, the relative pressure at point B presents a U-shaped distribution (shown in Table 2). The inflection point B of the isotherm was obtained from the curvature curves of the adsorption isotherm of the coal samples of different coal ranks shown in Fig 3 and then compared with the above analysis results, so as to accurately obtain the coordinates of point B. The ratio of the adsorption capacity at point B of the coal samples with different coal ranks to the total adsorption capacity in descending order is PH > CV > JB > LH > HG > YW. Low-rank coal (PH coal sample) accounts for the highest proportion of 31.24%. In comparison, the high-rank coal sample (YW coal sample) is the lowest, accounting for only 11.52%, indicating that the proportion of N_2_ molecules in the PH coal sample in the form of micropore filling and monolayer adsorption is higher than that in the other coal samples.

**Table 2:** N_2_ adsorption/desorption isotherm analysis of coal samples with different coal ranks.

Sample	*P/P*_*0*_ at point B	Percentage of *Q*_*B*_/%	Isotherm type	Hysteresis loop type	Pore shape
**PH**	0.09886	31.24	Ⅱ b	H2	Ink bottle hole
**JB**	0.06005	13.70		H3	Nonuniform slit hole
**LH**	0.04309	13.34			
**HG**	0.06226	11.77			
**CV**	0.11094	19.57	Ⅰ b	H4	Uniform and narrow slit holes
**YW**	0.16089	11.52			

Multilayer adsorption means that as the pressure increases, in addition to the adsorption of the first layer that is in direct contact with the surface of the adsorbent, a superposition of multilayer adsorption occurs successively, and the number of adsorption layers is infinite at the saturated vapor pressure. In the middle *P/P*_*0*_ and high *P/P*_*0*_ regions (low potential energy region), as the pressure increases, the gas molecules are more likely to undergo multilayer adsorption and capillary condensation in the mesoporous and macroporous regions [16]. In Fig 2, the coal samples with the six different coal ranks exhibit capillary aggregation hysteresis, which is mainly related to the shape of the internal pores of the solid, experimental temperature, and adsorption system. Except for the PH coal sample, the adsorption capacity of the other five coal samples increased significantly when 0.8 < *P/P*_*0*_ < 0.995, and unlimited multimolecule adsorption occurred. The adsorption isotherm increased sharply when the *P/P*_*0*_ approached 1. This shows that the gas molecules are adsorbed in the macropores of the coal, consistent with the results of the aforementioned monolayer adsorption analysis.

### Critical characteristics of micropore filling in low-pressure area

From the above analysis, due to the presence of strong adsorption sites with electron transfer interactions on the microporous surface, such as functional groups and acid sites, the adsorption potential is much greater than that on a flat surface. When micropore adsorption occurs, the gas molecules accumulated at the entrance of the micropore diffuse gradually into the pores. However, the potential fields of two adjacent pore walls in the micropore overlap with the gas molecules, and the force on the gas molecules is higher than that on the mesopores and macropores. The gas molecules are more strongly adsorbed, and the adsorption capacity rises sharply at a lower relative pressure. Then, the N_2_ adsorption isotherm is used to further analyze the critical state of the micropore filling of N_2_ molecules in the different rank coals.

#### Critical pressure for micropore filling in coals

**Fig 4:**
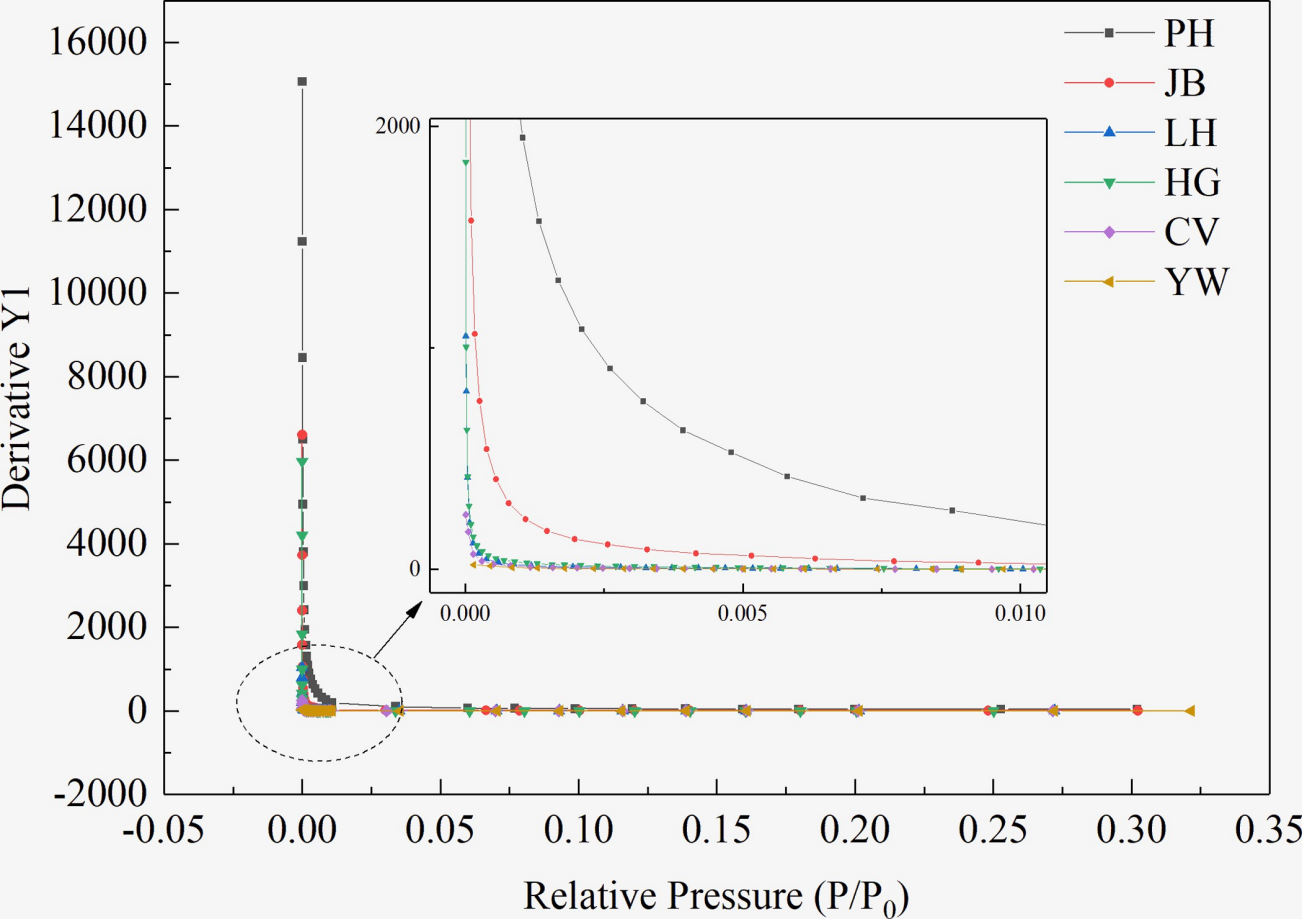
Differential curves of the adsorption isotherms of coal samples of different coal ranks.

Because micropore filling occurs under a very low relative pressure, the micropore filling is completed within a certain pressure range to enter the monomolecular layer adsorption or multilayer adsorption. Hence, as shown in Fig 4, the adsorption isotherms of the six coal samples with different coal ranks are differentiated to obtain the critical pressure range for the pores in the coals to complete the micropore filling, that is, the critical filling pressure (CFP).

From low to high coal rank, the CFP changes accordingly. The N_2_ molecules in low-rank coals (PH and JB coal samples) are filled with micropores in the *P/P*_*0*_ range of 1E-4–0.03, whereas the N_2_ molecules in the medium- or high-rank coals (LH, HG, CV, and YW coal samples) gradually complete micropore filling in the *P/P*_*0*_ range of 1E-4–0.01, as listed in Table 3.

**Table 3:** Differential curve analysis of the adsorption isotherms of different rank coal samples.

Sample	CFP (*P/P*_*0*_*)*	CPVD/nm	DA index
**PH**	8E-4–0.0339	1.73–2.19	1.3620
**JB**	5.5E-4–0.0302	1.70–2.17	1.5343
**LH**	2.4E-4–0.0100	1.64–1.99	1.5324
**HG**	1.4E-4–0.0104	1.61–2.00	1.8330
**CV**	5E-4–0.0106	1.69–2.00	2.5444
**YW**	8.4E-4–0.0107	1.73–2.01	2.4375

#### Critical pore size for micropore filling in coals

The DFT equation can be used to obtain the pore size distribution by solving the generalized adsorption isotherm (GAI) integral equation and to establish a correlation between the theoretical and experimental isotherms. This equation is expressed as follows:

$$N_{exp}(\frac{P}{P_{0}})=\int_{\omega_{min}}^{\omega_{max}}N_{theo}(\frac{P}{P_{0}},\omega )f(\omega )dw$$ (1)

where $N(\frac{P}{P_{0}})$ represents the adsorption isotherm data for the experiment, *ω* is the pore width, $N(\frac{P}{P_{0}},\omega )$ is the adsorption isotherm with a single hole width *ω*, and *f*(*ω*) is the pore size distribution function.

**Fig 5:**
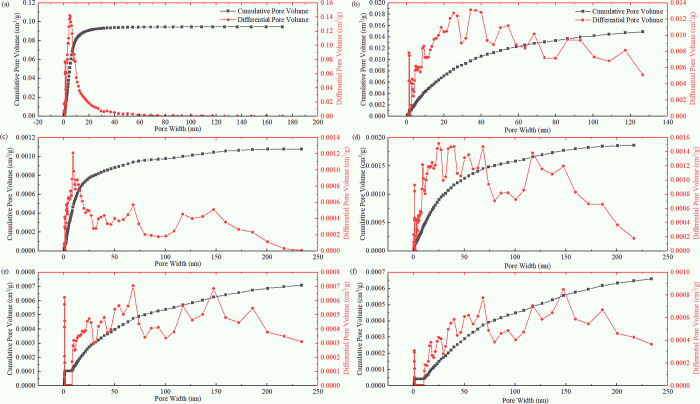
Cumulative pore volume and differential pore volume diagram of coals. (a) PH Sample, (b) JB Sample, (c) LH Sample, (d) HG Sample, (e) CV Sample, (f) YW Sample.

As shown in Fig 5, the pore size distributions of the different coal samples are quite different; the pore size distribution range is 0.46–233 nm. The pore size distributions of the PH and the LH coal samples are relatively simple, containing several 5 and 9 nm pores, respectively. The pore size of the other four types of coal samples is distributed in a wide range.

Dubinin and Astakhov proposed the Dubinin–Astakhov (DA) equation with empirical parameter n to describe the adsorption isotherm of micropore filling, highlighting the surface heterogeneity. The DA index n is considered a measure of the heterogeneity. As the degree of activation decreases, its value tends to be unified. It can be reasonably assumed that the DA equation index n qualitatively reflects the degree of heterogeneity of a material (pore size and surface chemistry) [29]. The parameters in the DA equation independently characterize the microporous structure and adsorption characteristics of an adsorbent, revealing the relationship between the microporous structure of the adsorbent and its adsorption performance, expressed in Eqs (2) or (3):

$$Q=Q_{0}e^{-({\frac{A}{\beta E_{0}})}^{n}}$$ (2)

$$logQ=logQ_{0}-{\left(2.3026\frac{RT}{\beta E_{0}}\right)}^{n}{\left(log\frac{P_{0}}{P}\right)}^{n}$$ (3)

where *Q* is the adsorption capacity at the relative pressure *P*/*P*_*0*_; *Q*_*0*_ is the limit adsorption capacity, which is generally considered to be the micropore volume; *A* is the adsorption potential energy; *β* is the affinity coefficient, expressed in Eq (4):

$$\beta =\frac{E}{E_{0}}$$ (4)

where *E* is the characteristic adsorption potential energy of the adsorbate gas; *E*_*0*_ is the characteristic adsorption potential energy of the standard adsorbate gas.

**Fig 6:**
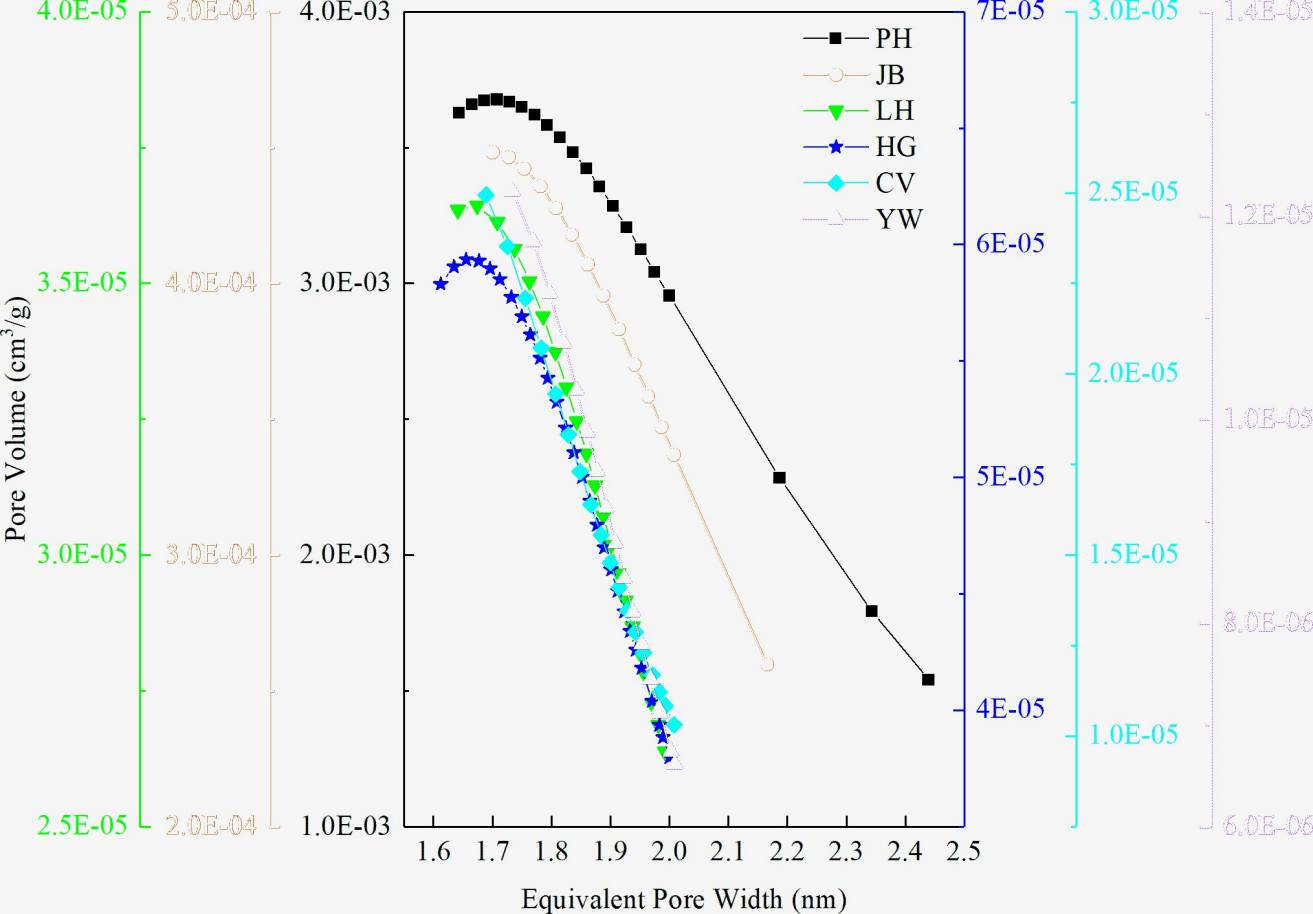
DA model analysis of the pore size distribution of the coal samples in the critical filling pressure range.

The DA equation was used to calculate the pore size distribution of the six coal samples with different coal ranks under CFP, and the critical pore size range (CPVD) for micropore filling was determined, as shown in Fig 6.

The CPVD of the six coal samples of different coal ranks is in the range of 1.61–2.19 nm; for the PH and JB coal samples, the range is 1.7–2.19 nm, and for the other four coal samples, the range is 1.61–2.00 nm, as listed in Table 3.

**Fig 7:**
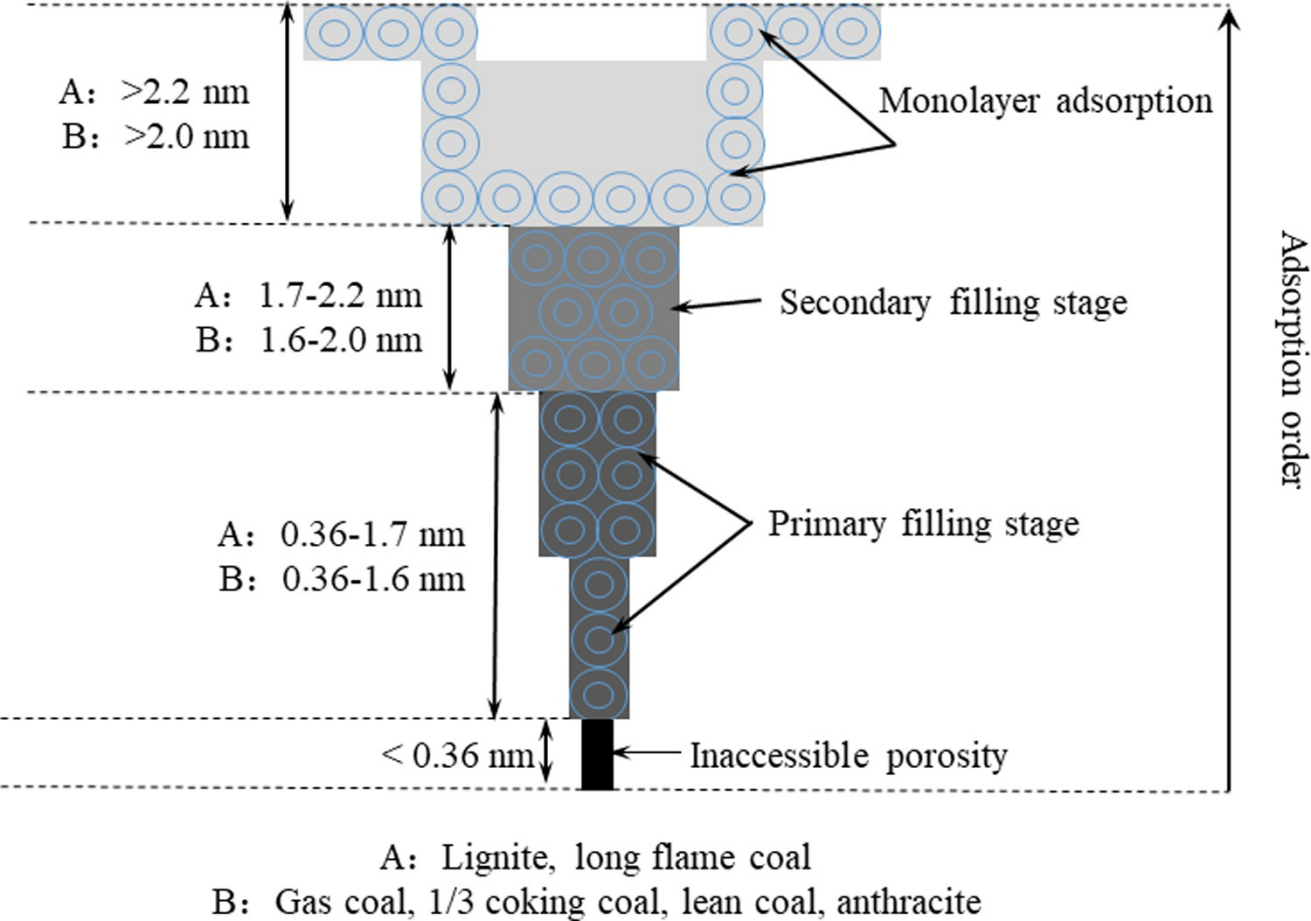
Main stages in the continuous filling of micropores during the adsorption process.

As shown in Table 3, there is a U-shaped correlation between the CFP, CPVD, and coal rank, that is, the higher the coal rank, the overall CFP and CPVD decrease first and then increase; the CPVD and CFP also have the same trend. Therefore, the primary and secondary filling stages are divided on the basis of the strength of the adsorption potential, and pores with a size less than 0.36 nm are called noncontact pores (based on the N_2_ molecular dynamics diameter of 0.364 nm). The transition of the micropores in coals with different coal ranks in different stages during the adsorption process is divided in Fig 7.

In addition, the DA index n can qualitatively reflect the degree of material heterogeneity. The value of n represents the filling rate of the micropores, the heterogeneity of the micropores, and the energy distribution. The DA index n of the six coal samples shows an increasing trend with increasing coal rank, as listed in Table 3, that is, PH < JB ≈ LH < HG < YW < CV. This shows that the micropore filling rate of the N_2_ molecules in low-rank coals is higher than those of the N_2_ molecules in the medium- and high-rank coals, the heterogeneity of the micropores is also stronger, and the energy distribution is wider.

#### Verification of the critical pore size for micropore filling

Three widely used models were selected to verify and discuss the analysis results described above, that is, the DA equation of the micropore filling theory (Eq (3)), Langmuir adsorption equation describing monolayer adsorption (Eq (5)), and the BET equation (Eq (6)) for multimolecular layer adsorption:

$$\frac{P}{P_{0}}\times \frac{1}{Q}=\frac{1}{BQ_{m}}+\frac{P}{P_{0}}\times \frac{1}{Q_{m}}$$ (5)

$$\frac{1}{Q(\frac{P_{0}}{P}-1)}=\frac{1}{Q_{m}C}+\frac{C-1}{Q_{m}C}\times \frac{P}{P_{0}}$$ (6)

Here, *Q* is the adsorption volume of nitrogen, *Q*_*m*_ is the monolayer saturated adsorption volume of nitrogen, *Q*_*0*_ is the pore volume of the coal sample, *P/P*_*0*_ is the relative pressure, *B* is the adsorption energy constant, and *C* is the BET constant related to the net heat of adsorption.

**Fig 8:**
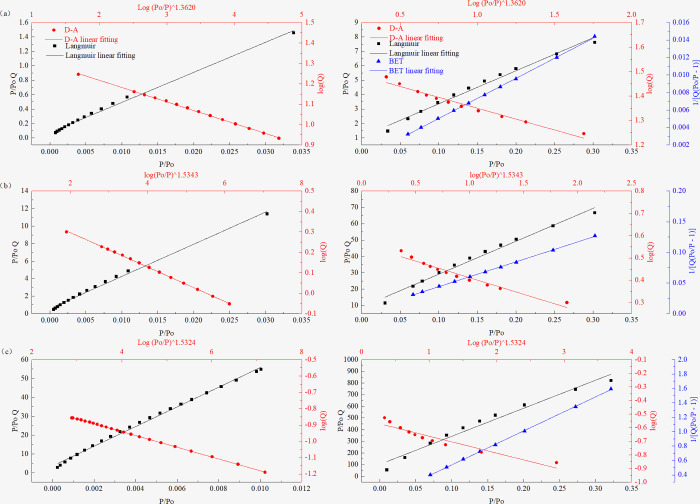
Fitting diagrams of coal samples of different coal ranks obtained using different models at different pressure sections. (a) PH Sample, (b) JB Sample, (c) LH Sample. (Left: Langmuir and DA model, Right: Langmuir, DA, and BET models).

**Fig 9:**
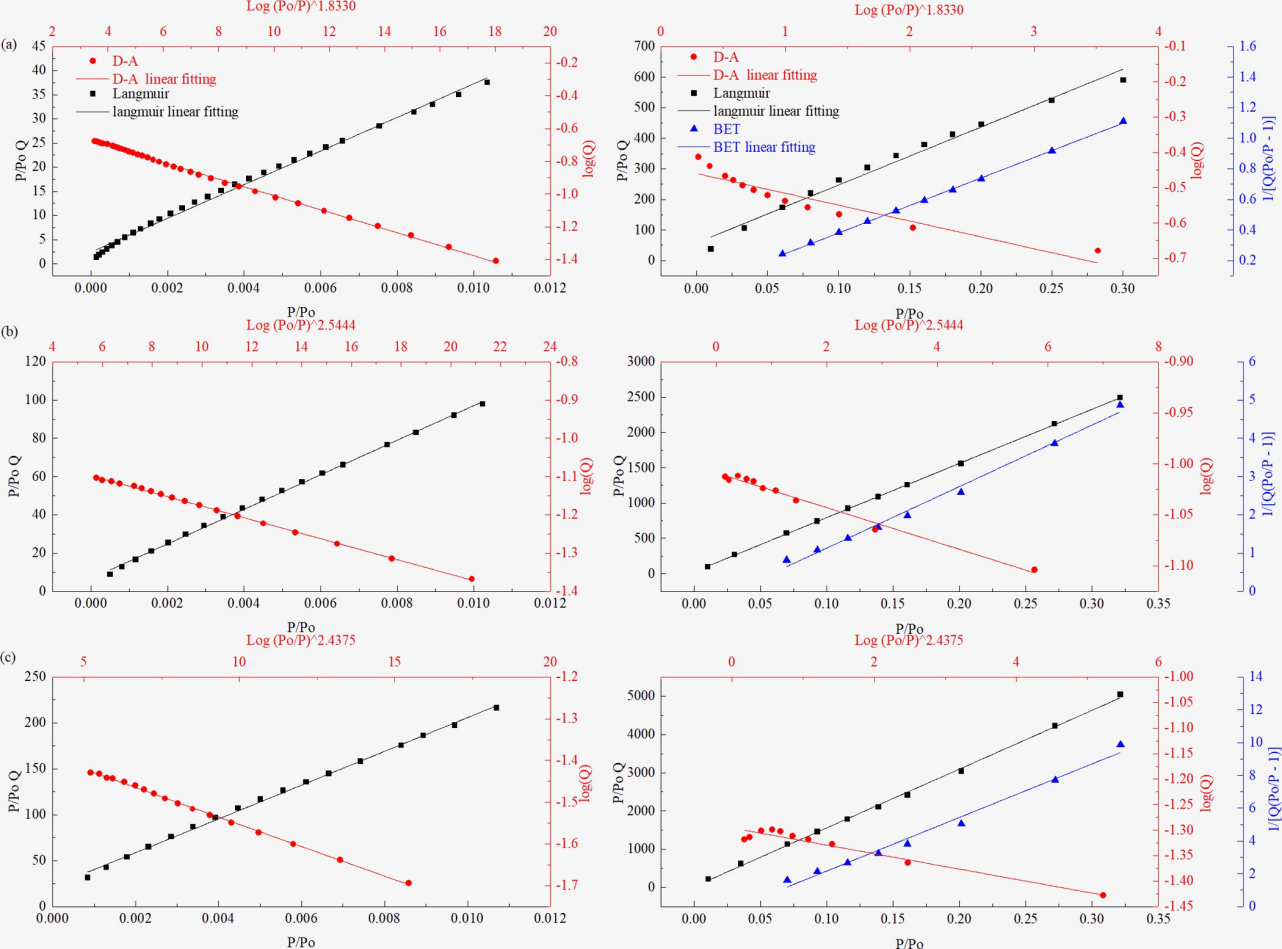
Fitting diagrams of coal samples of different coal ranks obtained using different models at different pressure sections. (a) HG Sample, (b) CV Sample, (c) YW Sample. (Left: Langmuir and DA model, Right: Langmuir, DA, and BET models).

After plotting *logQ* and *[log(P*_*0*_*/P)]* ^*n*^ in the DA equation, *P/P*_*0*_ *Q* and *P/P*_*0*_ in Langmuir equation, *1/[Q(P*_*0*_*/P-1)]* and *P/P*_*0*_ in the BET equation, the high linear correlation coefficients represent the characteristics of micropore filling, monolayer adsorption, and multilayer adsorption, respectively. According to the DA equation, when using *logQ* to plot *[log(P*_*0*_*/P)]* ^*n*^ in a certain pressure range, a straight line is obtained, and the degree of fit is relatively high, indicating that only micropore filling occurs in the above-mentioned pressure range. To verify the analysis results described in Sections “**Critical pressure for micropore filling in coals**” and “**Critical pore size for micropore filling in coals,**” the adsorption isotherm is divided into pressure ranges based on the CFP described in Section “**Critical pressure for micropore filling in coals**” and the applicable range of the BET equation (*P/P*_*0*_ = 0.05–0.35). A linear fitting is then performed, as shown in Figs 8 and 9. The figure on the left shows the fitting results of the DA and Langmuir equations in the critical pore filling pressure range, and the figure on the right shows the fitting results of the DA equation, Langmuir equation, and BET equation at *P/P*_*0*_ ≈ 0.01–0.35. Table 4 presents the fitting results.

**Table 4:** Analysis results of coal samples with different coal ranks obtained using different adsorption models in different pressure regions.

Sample	P/P_0_	Models		P/P_0_	Models		
		Langmuir	DA		Langmuir	DA	BET
**PH**	8E-4-0.0339	0.9942	0.99921	0.0339–0.3021	0.98522	0.96746	0.99976
**JB**	5.5E-4-0.0302	0.99402	0.99945	0.0302–0.30235	0.98547	0.95176	0.99954
**LH**	2.4E-4-0.0100	0.99619	0.99980	0.0100–0.3214	0.97082	0.8909	0.99972
**HG**	1.4E-4-0.0104	0.99503	0.99883	0.0104–0.3002	0.98249	0.87445	0.99956
**CV**	5.0E-4-0.0102	0.99865	0.99928	0.0102–0.3212	0.99987	0.98432	0.99027
**YW**	8.4E-4-0.0107	0.99772	0.99863	0.0107–0.3215	0.99855	0.93471	0.98462

Figs 8 and 9 show the analysis results of the six coal samples with different coal ranks fitted using different adsorption models. The linear fitting results show that the fitting accuracy of the DA equation is greater than that of the Langmuir equation in the CFP range (left of Figs 8 and 9). This indicates that micropore filling occupies a dominant position in this pressure range. In the pressure range after the micropore filling is completed (right of Figs 8 and 9), the equation fitting accuracy for the PH, JB, LH, and HG coal samples is BET equation > Langmuir equation > DA equation. This indicates that after the above coal samples have completed the micropore filling, the monolayer adsorption process is relatively short, mainly for multimolecule adsorption. For the CV and YW coal samples, the order is Langmuir equation > BET equation > DA equation. This shows that the N_2_ molecules in the two coal samples mainly undergo monolayer adsorption in a certain pressure range after the micropores are filled. The above analysis results can also be confirmed by combining the analysis of the inflection point of the isotherm shown in Section “**N**_**2**_ **adsorption/desorption isotherms.**” High-rank coals (CV and YW coal samples) require a higher pressure to reach the inflection point, indicating that the transition between the different adsorption forms of the high-rank coal samples is slower and that it requires a higher pressure. A high relative pressure completes monolayer coverage, while low-rank coal completes monolayer adsorption earlier than high-rank coal and enters the stage of multimolecular layer adsorption faster.

## Conclusions

From low to high coal rank, the N_2_ adsorption/desorption isotherms of coal samples transitioned from type II to type I, the number of micropores into which N_2_ molecules could enter increased, and the micropore filling process was relatively prolonged. Due to the intercalation phenomenon during the adsorption process, the distance between the layers was close to the micropores, and it was difficult for the N_2_ molecules to desorb after entering. The higher the coal rank, the more evident the deviation in the desorption curve.

Compared with the other five coal samples, the pore structure of lignite was relatively uniform, with ink bottle pores as the main component. The adsorption capacity for the N_2_ molecules was significantly higher than those of the other five coal samples. The filling was via micropores and monolayer adsorption. The proportion of N_2_ molecules was higher than those in the other five coal samples. Medium-rank and high-rank coals exhibited a wide range of pore size distribution, complex pore structure, and a large number of molecular-scale pores that could not be entered by the N_2_ molecules. Therefore, the adsorption capacities of the gas coal, 1/3 coking coal, lean coal, and anthracite measured with N_2_ as the adsorbent gas were much lower than those of lignite and long-flame coal.

The critical pressure and critical pore size for the micropore filling of N_2_ molecules in coals exhibited a U-shaped correlation with the coal rank. From low to high, the critical pressure and critical pore size decreased first and then increased. The transformation of the N_2_ molecules from microporous filling to single-layer adsorption in the low-rank coals required a high pressure, and the transformation from single-layer adsorption to multimolecular-layer adsorption in high-rank coals required a high pressure. Low-rank coals could complete single-layer adsorption before the high-rank coals and could transition to multimolecular layer adsorption.

In the LPGA-N_2_, the critical pore size for the micropore filling of N_2_ molecules in the coal samples of different ranks was found to be in the range of 1.61–2.19 nm; for lignite and long-flame coal, the range was 1.7–2.19 nm; for gas coal, 1/3 Coking coal, lean coal and anthracite, the range was 1.61–2.00 nm. The high-rank coals had a smaller critical filling aperture than the low-rank coals.

In the future, we will continue to explore the critical filling characteristics of different adsorbate gases in coal and further explore the occurrence mechanism and migration laws of coalbed methane.

## Supporting information

S1 File (ZIP)
